# Acquired Non-histaminergic Angioedema With C1q Autoantibody and Urticaria: A Case Report

**DOI:** 10.7759/cureus.43841

**Published:** 2023-08-21

**Authors:** Andrew P Kochvar, Gavin Cobb, Celina C Bernabe, Terry Levine

**Affiliations:** 1 College of Osteopathic Medicine, Kansas City University, Kansas City, USA; 2 Allergy and Immunology, Allergy & Asthma Care, P.A., Overland Park, USA

**Keywords:** inh-aae, bradykinin, aae, c1q autoantibody, autoimmune disease, icatibant, lanadelumab, urticaria, anti-c1q antibody, acquired angioedema

## Abstract

Acquired angioedema (AAE) is a rare disease with life-threatening complications. This pathology has classically been associated with medication use and B cell lymphoproliferative disorders. In this report, we describe a 61-year-old man with a six-year history of angioedema, unrelated to any known triggers or malignancy. Extensive workup has led to a diagnosis of idiopathic nonhistaminergic AAE with normal C1 inhibitor. The patient is currently being treated with lanadelumab, which has resolved the patient’s symptoms. This case provides insight into the onset, exploration, treatment, and outcomes of an extremely rare disease process.

## Introduction

Angioedema is defined as a well-demarcated swelling of the skin and subcutaneous tissues mediated by histaminergic or bradykinin pathways [1]. Broadly, clinical diseases of angioedema are categorized as either hereditary or acquired absences of key functional proteins such as the C1 inhibitor (C1-INH) of the complement pathway, leading to uncontrolled pathway activation and production of bradykinins [2-3].

Current estimates place the prevalence of AAE at approximately 1:100,000 to 1:500,000, though this is considered an underestimation [1]. The diagnosis of AAE should be explored in patients within the fourth decade with recurrent non-urticarial edema without identifiable triggers or a family history of angioedema [4].

AAE is divided into two major subclasses. AAE type I is often associated with B-cell proliferative disorders, which can accelerate the catabolism of C1-INH [4]. AAE type II is associated with autoantibodies against C1-INH [5]. The current standard for diagnosis combines laboratory findings and clinical criteria. Typical laboratory workup used with cases of suspected AAE includes quantitative C1-INH, C1-INH functional activity, C4, and C1q [3]. Low C1q levels are only seen in AAE versus normal levels associated with hereditary angioedema (HAE) [1,3]. Due to the reported association between AAE and underlying lymphoproliferative disorders, it may be appropriate in some cases to conduct a workup for underlying malignancies [1,3].

Additional subcategories of angioedema have recently been described as unique entities separate from those already mentioned. Patients with non-HAE disease who respond to anti-histamines are diagnosed with idiopathic histaminergic AAE (IH-AAE) versus non-histaminergic idiopathic AAE (InH-AAE) [3,6]. Here, we present a case of InH-AAE associated with anti-C1q antibody and low C1q levels, normal C1 esterase inhibitor levels, and an otherwise benign hematologic and rheumatologic workup that failed maximum antihistamine therapy and biologics targeted to the allergic cascade.

## Case presentation

A 56-year-old male presented to the allergy clinic with a history of recurrent spontaneous urticaria and angioedema initially identified three years prior without a known trigger. His family medical history was negative for angioedema, though he reported that first-degree relatives had histories of severe allergies and asthma. One year prior to the initial visit, the patient presented to a different allergy clinic with urticaria on the back, flanks, and lip without a known trigger. Initial allergic workup demonstrated allergy to tree pollens and low-grade reactions to other pollens. Thus, no further workup was done and he was placed on cetirizine. A year after this and one month prior to the presentation to our clinic, the patient had recurrent urticaria and one episode of angioedema of the tongue and pharynx which awoke the patient and prompted a visit to urgent care. He was placed on montelukast (leukotriene modulator), cetirizine (second-generation H1-antagonist), and ranitidine (H2 antagonist) without improvement.

On presentation to our clinic one month after this episode, physical exam was positive for diffuse urticarial lesions with apparent contact dermatitis of the lower extremities, moderate swelling, erythema of the nasal turbinates, and increased lymphoid tissue in the oropharynx.

Quinapril (angiotensin-converting enzyme (ACE) inhibitor), used for his existing hypertension, was discontinued as a potential source of bradykinin-mediated angioedema. Initial medication trial included the addition of fexofenadine (second-generation selective H1 antagonist), augmented betamethasone cream (topical corticosteroid), a course of oral prednisone (corticosteroid), and continuation of montelukast. He responded, but episodes of angioedema and spontaneous urticaria resumed days after finishing his steroid course, at which point he was reevaluated. Physical exam revealed diffuse urticaria of the palms of both hands and angioedema of the hands, lips, and nose. Over the next several months, he had frequent recurrences with angioedema of the face, oropharynx, uvula, pharynx, tongue, legs, hands, and feet. Throughout these presentations, step-ups of medication dosages and oral prednisone were attempted as temporizing measures while further workup was performed.

An extensive workup of this patient was performed, which led to a working diagnosis of AAE type II. Initial laboratory test results demonstrated hypocomplementemia with decreased C1Q, C3C, C4C, and CH50 with normal total C1-INH levels and function. Gamma globulins against Epstein-Barr virus (EBV) and cytomegalovirus (CMV) antigens were also elevated without definitive evidence of active infection. Hematology oncology consult ruled out lymphoproliferative disorders and porphyria as causal etiologies. Evaluation for infection with *Trypanosoma cruzi* was performed because the patient had recently lived in an area where Chagas disease is endemic; however, the result returned negative. Stool evaluation for ova and parasites also returned without positive findings. Rheumatologic labs were unremarkable for autoimmune comorbidities. Ultimately, Enzyme-Linked Immuno Sorbent Assay (ELISA) assays were sent and returned positive for elevated C1q autoantibodies and immune complexes present without C1-INH autoantibodies. Table 1 presents the full panel of relevant laboratory tests and Table 2 demonstrates persistent hypocomplementemia, normal C1-INH level and function, and low levels of C1q.

**Table 1:** Laboratory workup results Normal values used were taken directly from the patient chart EBV: Epstein-Barr virus; CMV: cytomegalovirus

Test	Result	Normal Value
C1 Esterase Inhibitor	31 mg/dl	21-39 mg/dl
C1 Esterase Inhibitor Function	100 %	>68%
C1 Esterase Inhibitor Autoantibody	26.5%	0-39%
Complement C1q	3.6 mg/dl	5.0-8.6 mg/dl
Immune Complex by C1q Binding	65.7 mcg eq/ml	<25.1 mcg eq/ml
Complement C3	59 mg/dl	90-180 mg/dl
Complement C4	7 mg/dl	16-47 mg/dl
CH50	29 U/ml	31-60 U/ml
C-Reactive Protein	2.34 mg/dl	<0.80 mg/dl
Anti-Nuclear Antibody (ANA)	Negative	Negative
Rheumatoid Factor	5 IU/ml	<14 IU/ml
Anti-Citrullinated Peptide IgG	<16 IU	<20 IU
Tryptase	5 ng/ml	<11 ng/ml
Galactose Alpha-1,3-galactose IgE	<0.10 kU/L	<0.35 kU/L
Total IgE	508 kU/L	<114 kU/L
EBV Viral Capsid IgM (VCA)	<36 (U/ml)	<36 (U/ml)
EBV Viral Capsid IgG (VCA)	378 (U/ml)	<18 (U/ml)
EBV Nuclear Antigen IgG (EBNA)	468 (U/ml)	<18 (U/ml)
CMV IgG	6.60 U/ml	<0.60 (U/ml)
CMV IgM	<30 AU/ml	<30 Au/ml

**Table 2:** Trended laboratory values for C1-INH, C1q, C3, and C4 throughout the patient's disease course Normal values used were taken directly from the patient chart C1-INH: C1 inhibitor

	2021	2018	2017	Normal Values
C1 Esterase Inhibitor Functional	92%	100%	100%	>68%
C1 Esterase Inhibitor, Protein	24 mg/dl	31 mg/dl	25 mg/dl	21-39 mg/dl
C1q	<3.6 mg/dl	<3.6 mg/dl	3.6 mg/dl	5.0-8.6 mg/dl
Complement C3	61 mg/dl	78 mg/dl	59 mg/dl	82-185 mg/dl
Complement C4	8 mg/dl	8 mg/dl	7 mg/dl	15-53 mg/dl

**Figure 1:**
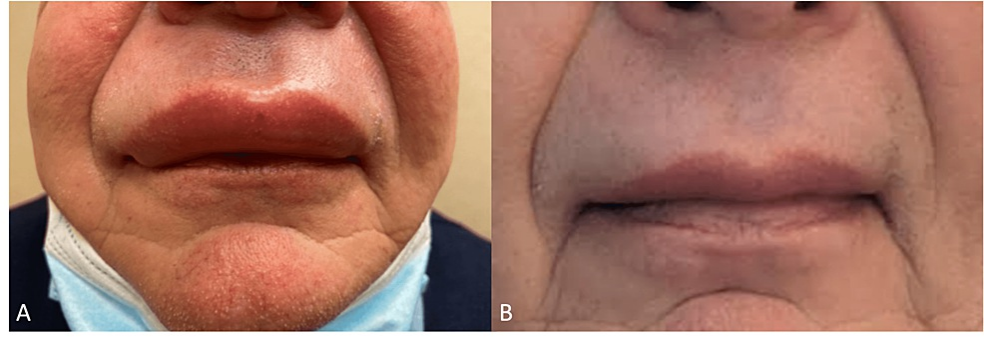
Angioedema attack of the upper lip and nares before (A) and after (B) icatibant administration

Throughout his workup, he was treated with four-times daily dosing of cetirizine followed by a trial of fexofenadine. Ranitidine was added twice daily without improvement. An extended trial of omalizumab (monoclonal antibody against IgE) was added to this regimen without symptomatic relief despite significant overlap with the antihistamine regimen. Once a definitive diagnosis of InH-AAE was made, the patient was trialed on subcutaneously administered lanadelumab (monoclonal antibody against plasma kallikrein) every four weeks for angioedema prophylaxis and given icatibant (synthetic selective bradykinin B2 receptor antagonist) as an on-demand rescue agent (Figure 1). This regimen significantly reduced the number and severity of angioedema and urticaria episodes from daily to three to four times per week. Symptoms persisted despite monthly use. Thus, the lanadelumab dose frequency was increased to every two weeks.

## Discussion

The present case demonstrates an atypical presentation of InH-AAE with urticaria and hives, which was unresponsive to antihistamines and anti-IgE biologics. Hives and urticaria are atypical of angioedema disease in both HAE and AAE [3]. InH-AAE is a poorly studied entity, though case series specific to this entity report facial swellings as the most common location of angioedema and, to a lesser degree, upper airway involvement and abdominal symptoms [7].

Historically, the patient has never responded to agents targeted to the allergic cascade but has responded well to those targeting elements of the contact-kinin system, which are FDA-approved to treat HAE but not AAE [8,9]. Despite this, multiple cases of AAE have been successfully treated with lanadelumab [10-12].

Improvement with icatibant administration has been described as the most specific test for bradykinin-mediated angioedemas, particularly in patients with InH-AAE after a trial of four-times daily antihistamine dosing and omalizumab fail to improve symptoms [13]. Daily administration of tranexamic acid has also been explored as a prophylactic treatment for refractory AAE with good response rates [13-14]; however, this has not been trialed in our patient.

AAE can be associated with comorbid conditions, which must be ruled out with an extensive workup of an individual’s disease. Despite a negative workup for diseases associated with AAE, namely B-cell lymphoproliferative disease, the possibility that symptomatology may be prodromal cannot be excluded, though this association is unlikely because cases of AAE typically involve aberrations of C1-INH level or function, rather than C1q [15-16].

The presence of C1q antibody has been demonstrated in other potential comorbid disease processes, including but not limited to HAE, lupus nephritis, and hypocomplementemic urticarial vasculitis [17-19]. However, clinical and laboratory evidence of such differential diagnoses in this patient has been insufficient to support an alternative diagnosis thus far.

## Conclusions

This case illustrates the gaps in knowledge that remain about this unique pathology. Angioedema occurs through various vasoactive mediators and can be further delineated through several characteristics. Due to the rare presentation of this patient and the relative scarcity of research into the ideal treatment modalities, this patient had to undergo multiple trials of therapy before determining a final diagnosis and therapy for his condition. Our case elucidates the necessity of further research into the more rapid diagnosis of InH-AAE with C1q autoantibody and fine-tuning of the treatment algorithm to improve patient quality of life and prevent negative outcomes.
